# Gut Microbiota Exceeds Cervical Microbiota for Early Diagnosis of Endometriosis

**DOI:** 10.3389/fcimb.2021.788836

**Published:** 2021-12-07

**Authors:** Liujing Huang, Bingdong Liu, Zhihong Liu, Wanqin Feng, Minjuan Liu, Yifeng Wang, Dongxian Peng, Xiafei Fu, Honglei Zhu, Zongbin Cui, Liwei Xie, Ying Ma

**Affiliations:** ^1^ Department of Obstetrics and Gynecology, Zhujiang Hospital, Southern Medical University, Guangzhou, China; ^2^ Guangdong Provincial Key Laboratory of Microbial Culture Collection and Application, State Key Laboratory of Applied Microbiology Southern China, Institute of Microbiology, Guangdong Academy of Sciences, Guangzhou, China; ^3^ Department of Psychiatry, The First Affiliated Hospital of Jinan University, Guangzhou, China; ^4^ College of Public Health, Xinxiang Medical University, Xinxiang, China

**Keywords:** endometriosis, gut microbiota, peritoneal fluid, *Lachnospiraceae Ruminococcus*, *Pseudomonadaceae Pseudomonas*

## Abstract

The diagnosis of endometriosis is typically delayed by years for the unexclusive symptom and the traumatic diagnostic method. Several studies have demonstrated that gut microbiota and cervical mucus potentially can be used as auxiliary diagnostic biomarkers. However, none of the previous studies has compared the robustness of endometriosis classifiers based on microbiota of different body sites or demonstrated the correlation among microbiota of gut, cervical mucus, and peritoneal fluid of endometriosis, searching for alternative diagnostic approaches. Herein, we enrolled 41 women (control, n = 20; endometriosis, n = 21) and collected 122 well-matched samples, derived from feces, cervical mucus, and peritoneal fluid, to explore the nature of microbiome of endometriosis patients. Our results indicated that microbial composition is remarkably distinguished between three body sites, with 19 overlapped taxa. Moreover, endometriosis patients harbor distinct microbial communities versus control group especially in feces and peritoneal fluid, with increased abundance of pathogens in peritoneal fluid and depletion of protective microbes in feces. Particularly, genera of *Ruminococcus* and *Pseudomonas* were identified as potential biomarkers in gut and peritoneal fluid, respectively. Furthermore, novel endometriosis classifiers were constructed based on taxa selected by a robust machine learning method. These results demonstrated that gut microbiota exceeds cervical microbiota in diagnosing endometriosis. Collectively, this study reveals important insights into the microbial profiling in different body sites of endometriosis, which warrant future exploration into the role of microbiota in endometriosis and highlighted values on gut microbiota in early diagnosis of endometriosis.

## Introduction

Endometriosis (EM) is a chronic inflammatory gynecological disease, with a high prevalence (~10%) of reproductive-age women, and affects approximately 176 million women worldwide (Giudice and Kao, 2004; Hickey et al., 2014). Defined as the implantation of endometrial-like tissue outside the uterus, the ectopic endometrium is commonly found in the pelvis. EM is associated with high morbidities of pelvic pain, dysmenorrhea, dyspareunia, and infertility (Nnoaham et al., 2011). The relative symptoms of EM are not exclusive, giving rise to the delay of diagnosis typically by 8–10 years (Greene et al., 2009). For many women, simply having a diagnosis of EM brings relief. Laboratory tests, ultrasonography, and magnetic resonance imaging (MRI), being parts of the initial battery of investigations, can only provide auxiliary diagnosis (Gupta et al., 2000). In order to establish a criterion-standard diagnosis and ease the debilitating syndrome, a minimally invasive laparoscopy operation will be adopted to confirm and remove the ectopic endometrium (Kiesel and Sourouni, 2019). However, patients who received primary surgery showed a high recurrence rate about 40%–45% and would engender further surgery (Chen et al., 2018), while the excision of endometrioma may elicit temporary detrimental effects on ovarian reserve (Garry, 2004; Goodman et al., 2016). To date, the pathogenesis of EM remains elusive, with current findings suggesting a multifactorial mechanism. One of the most broadly accepted hypotheses is proposed in 1927 by Sampson, suggesting that retrograde menstruation through fallopian tubes backs up into the peritoneal cavity followed by colonization of the endometrial tissue (Sampson, 1927). In addition, other theories suggest that fragmented endometrial tissue may migrate and implant through blood or lymphatic circulation, and hormone-induced metaplasia (Sampson, 1927; Rösner and Böger, 1979). Genetics, environmental factors, and immune system are also known to be correlated with the development of EM (Giudice and Kao, 2004). However, none of the theories have elucidated the pathogenesis of EM, indicating that unknown modulators may present during disease progression. Most importantly, 80% women at reproductive age experience retrograde menstruation, but the prevalence of EM is around 10%, giving us a hint that the microenvironment of peritoneal cavity may be different between EM patients and women without EM.

In the traditional concept, the upper genital tract and peritoneal cavity have been largely considered to be sterile areas. Recently, this concept has been challenged by a considerable number of studies investigating microbial composition across the human body (Chen et al., 2017; Ata et al., 2019). Microbial seeding within the peritoneal cavity may originate from three main routes, which include the lymphatic system, bloodstream, and female genital tract (Stinson et al., 2017; Baker et al., 2018). In the past decades, we have reached the concept that the microbiome plays a key role in maintaining human health. Dysbiosis can perturb the immunomodulation of the host and result in the development of several inflammatory diseases (Blaser and Blaser, 2014). With the rapidly developed technologies used in the sequencing of the human genome, a wide range of analytical tools are used to assess biological parameters, which are defined as biomarkers (Atkinson et al., 2001). If so, a desirable goal would be to seek for objective biomarkers for EM diagnosis at an early stage without surgical operation. Although a direct contact between the gut microbiota and EM has not been proven, studies on the pancreas have demonstrated that gut flora can migrate into the pancreas and influence the pancreatic microenvironment (Pushalkar et al., 2018), giving us a hint that the same pattern may exist for EM. Women with EM are at an elevated risk of developing depression and anxiety disorders, which are linked to the alteration of microbiome (Neufeld et al., 2014), compared to those without EM (Chen et al., 2016). There are increasing investigations on the correlation between microbiome and EM of murine models and women, which have provided preliminary evidence of the bidirectional effect between EM and microbiota as well (Yuan et al., 2018; Ata et al., 2019).

Up to now, the composition and variation of microbes in and on the human body remain uncertain. In a retrospective study researching the comparison of vaginal, cervical, and gut microbiota between women with stage 3/4 EM and healthy controls, a complete absence of genera of Atopobium in the vagina and cervix and Shigella/Escherichia-dominant stool in EM patients was observed (Ata et al., 2019). Nevertheless, serum cancer antigen 125 (CA125), neutrophil-to-lymphocyte ratio (NLR), cytokines, etc., were found to be potential diagnostic factors for EM (Muyldermans et al., 1995; May et al., 2010; O et al., 2018). These observations add importance to a possible infectious etiology in EM. However, few auxiliary diagnosis methods emerged from such analysis and either of the previous studies has demonstrated a predictive model with potential to separate EM patients from the control group.

Thus, to address the confounding variables associated with EM, we initially recruited women with EM relative symptoms under strict inclusion and exclusion criteria and combined 16S rRNA gene sequencing and manifold forms of bioinformatic analysis trying to solve the puzzle for the first time. The results show that microbial signatures of the gut and peritoneal cavity of EM individuals significantly differ from those of the controls, whereas the cervix harbors a low-density and low-richness microbiome and is dominated by *Lactobacillaceae Lactobacillus*. Moreover, we demonstrated the microbial network and detected specific fecal microbiota that were correlated with *P. Pseudomonas* in peritoneal fluid. Overall, this study comprehensively characterizes the microbiome of the gut, cervix, and peritoneal cavity in the control group and EM patients and elucidates the potential of microbial markers as non-invasive diagnostic tools for identifying EM patients from the control group. The bioinformatic analysis of the gut microbiome has provided stirring glimpses into the putative role of the gut microbiota in directing personalized medical therapy and patient management.

## Results

### Characterization of Participants

**Figure 1:**
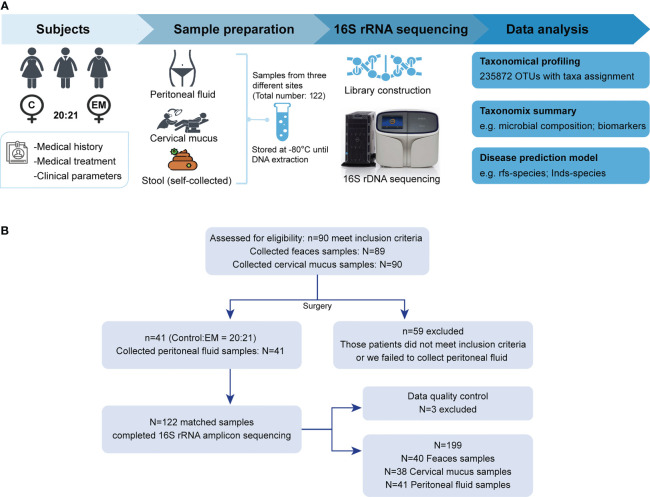
Study design and participant screening. **(A)** Overview of the study design. **(B)** The flow scheme of participant enrollment.

Participants in this study were recruited in the Gynaecology and Obstetrics Department of Zhujiang Hospital (**Figure 1A**). Screening and enrollment commenced on June 5, 2019, and the last participant was discharged on October 28 2019. Two rounds of screening were performed before and after the surgery (**Figure 1B**). The criteria of participant inclusion and exclusion were outlined below. Those participants, suspected to have benign gynecology disease, were submitted to laparoscopy. Participants were assigned to the endometriosis group after confirmation by laparoscopy together with biopsy analysis. Women in the control group were confirmed not to have endometriosis. All participants in this study neither had a record of receiving hormonal therapy or antibiotics in the past 3 months nor had abdominal surgery history or been diagnosed with autoimmune, inflammatory, and/or neoplastic diseases. Eventually, we admitted 20 women with benign gynecological disease as the control group and 21 EM patients and obtained samples from three different body sites of each participant. The recruited cohort was aged 36.2 ± 9.52 years (control group: 34.0 ± 10.8 years old, EM: 38.3 ± 7.88 years old) with a body mass index of 22.9 ± 6.13 kg m^−2^ (control group: 24.3 ± 8.16 kg m^−2^, EM: 21.5 ± 2.79 kg m^−2^; **Table 1**). Detailed clinical parameters were presented in supplemental materials (**Supplemental Table 1**). We further classified all samples into six groups (FN, feces of the control group; FE, feces of EM; CVN, cervical mucus of the control group; CVE, cervical mucus of the EM; PFN, peritoneal fluid of the control group; PFE, peritoneal fluid of the EM), based on the sources of microbial samples of different health conditions. Finally, 122 samples were successfully obtained, followed by 16S rRNA gene sequencing. Three samples with less than 10,000 reads were excluded. Data from the rest 119 well-matched samples, including feces, cervical mucus (CV), and peritoneal fluid (PF), were used to characterize microbial signatures between the control group and EM subjects.

**Table 1:** Baseline clinical characteristics between the control group and EM group.

Parameters	Control (n= 20)	EM (n = 21)	*p*-value
Hospital length of stay (day)	5.10 ( ± 1.59)	5.71 ( ± 1.59)	0.222
Age (year)	34.0 ( ± 10.8)	38.3 ( ± 7.88)	0.161
Height (cm)	157 ( ± 15.7)	159 ( ± 5.19)	0.522
Weight (kg)	57.8 ( ± 11.2)	54.6 ( ± 8.29)	0.301
BMI (kg m^-2^)	24.3 ( ± 8.16)	21.5 ( ± 2.79)	0.154
Glucose (mmol L^-1^)	4.37 ( ± 0.85)	4.55 ( ± 1.40)	0.622
WBC (×10^9^ L^-1^)	7.05 ( ± 1.83)	6.60 ( ± 1.85)	0.435
Neutrophils%	61.5 ( ± 7.69)	64.1 ( ± 10.3)	0.359
Lymphocyte%	30.3 ( ± 6.78)	29.3 ( ± 10.6)	0.715
Eosinophil%	1.82 ( ± 1.43)	1.49 ( ± 1.17)	0.412
Monocyte%	6.15 ( ± 1.89)	5.86 ( ± 1.82)	0.622
Baso%	0.22 ( ± 0.21)	0.20 ( ± 0.18)	0.685

BMI, body mass index; WBC, white blood cell; Baso, basophilic granulocyte.

Data were expressed as mean ± SD. p-value denotes two-tailed Student’s t-test.

### Microbiota Differs Between Gut, Cervical Mucus, and Peritoneal Fluid

**Figure 2:**
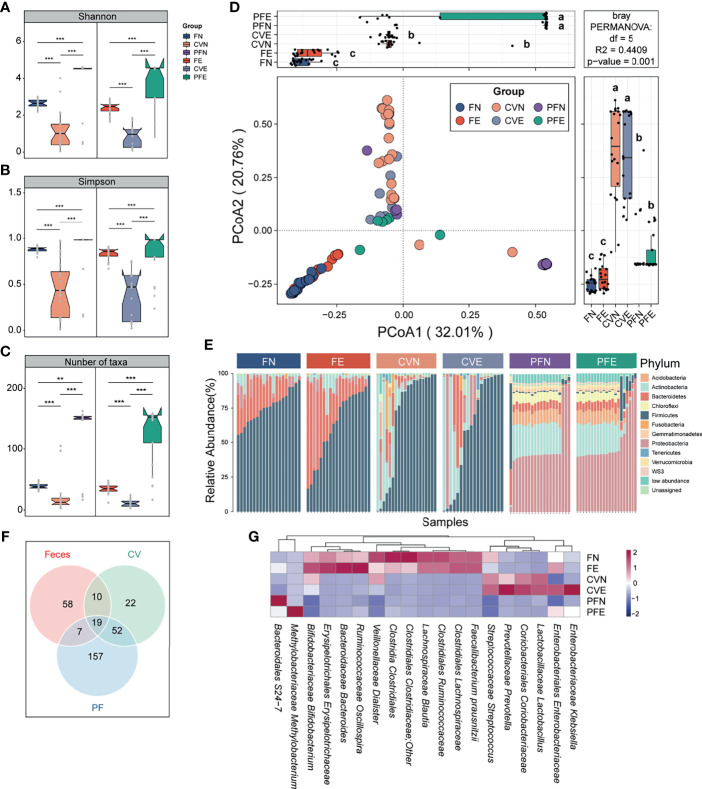
Microbiota composition was distinct between different body sites. **(A–C)** Comparison of the α diversity based on Shannon diversity, Simpson index, and number of taxa after decontamination in different body sites of controls and EM subjects. Statistical significance was determined by the Mann–Whitney U test. **p < 0.01, ***p < 0.005. **(D)** The PCoA was assessed using the Bray–Curtis dissimilarities matrix based on microbiota at the species level (mean relative abundance > 0.1%; prevalence > 10% individuals). Different letters (a, b, c) indicate significant differences (p < 0.05) between six groups according to the least significant difference (LSD). Changes in microbiome composition were assessed with permutational analyses of variance (PERMANOVA, R2 = 44.09%, p = 0.001), indicating that the largest variance in the microbiota was at the body sites level. **(E)** Cumulative bar charts of the most abundant taxa at the phylum level. Samples are ordered according to increasing relative abundances of the most abundant phylum within each group. **(F)** Venn diagram shows the number of commensal bacteria across different body sites. **(G)** Heatmap displays 19 taxa shared by different body sites.

The diversity (Shannon index) and richness (Simpson index) of bacterial complexity were examined and compared between feces, CV, and PF under different health conditions. Both control group and EM group showed higher richness and diversity in PF and feces compared to CV (**Figures 2A, B**). After data decontamination (taxa with mean relative abundance < 0.1% or prevalence < 10% within each group were discarded as potential contaminants), the remaining taxa count in FN, CVN, PFN, FE, CVE, and PFE were 38.9 ± 4.88, 21.6 ± 28.0, 127 ± 54.0, 35.0 ± 8.38, 11.4 ± 6.2, and 126 ± 48.5 (**Figure 2C**; mean ± SD), respectively, from which we can draw a conclusion that PF harbors the highest number of taxa while CV has the lowest biomass and diversity of microbiome. Taken together, we found distinct microbial complexity and communities in the gut and peritoneal fluid, differing from that of the cervix. Samples from different body sites were subjected to principal coordinate analyses (PCoA) based on Bray–Curtis distances and were grouped into distinct clusters. PCoA of species revealed significant differences between the microbiota composition in different body sites of patients with EM and that of controls (least significant difference, different letters denote statistical significance; **Figure 2D**). PERMANOVA power estimation was performed to assess the effect of the grouping factor upon the sampled microbiome (R2 = 0.4409, p = 0.001).

Continuing to probe into the variations corresponding to the control group and EM group in different body sites, we compared the bacterial abundance between groups at the phylum level. The top 10 most abundant phyla in each group were presented in the cumulating bar plot. Among them, the most abundant phylum in stool included *Firmicutes*, *Bacteroidetes*, and *Proteobacteria*, all with prevalence >90%. The most abundant phylum in CV was *Firmicutes*, *Actinobacteria*, and *Proteobacteria*. As for PF samples among subjects, *Proteobacteria*, *Actinobacteria*, *Acidobacteria*, and *Bacteroidetes* appeared to be dominant with prevalence >80%. Among individual phylum-level features profiled across three body sites, none of the presented phyla segregated significantly between the control group and EM group (**Figure 2E**). Moreover, cross-body site comparisons were explored by determining the taxa shared between groups (**Figure 2F**) and presenting the average relative abundance of those bacteria harbored by different habitats (**Figure 2G**). This approach allowed us to ascertain that the microbial profile of the cervical mucus and peritoneal fluid were similar as alluded to previous studies and identify 19 taxa presented in all three different body sites.

### Fecal Microbiota Significantly Differs Between Control Group and EM Group

**Figure 3:**
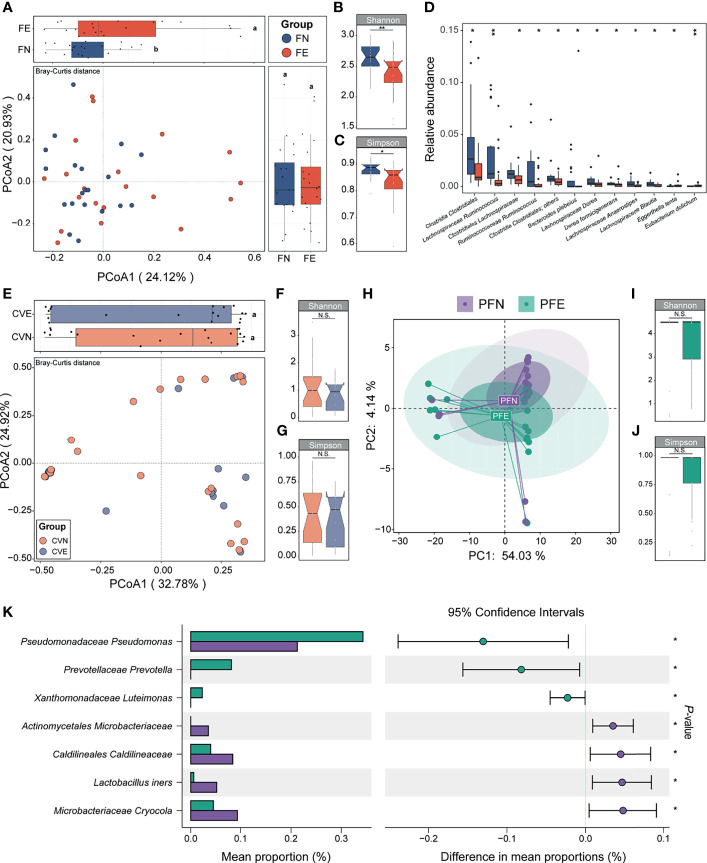
Between-group and within-group microbial community differences in control and EM individuals. **(A)** Fecal microbial community structural difference between controls and EM individuals by PCoA plotting based on Bray–Curtis dissimilarity data sets at the species level. Different letters (a, b) indicate significant difference (p < 0.05) between groups according to the least significant difference (LSD). **(B, C)** Differences in alpha-diversity between the control clusters from EM. **(D)** Box plots illustrated the relative abundance of differential bacteria between groups; box boundaries show quartile. **(E)** Cervical mucus microbial community structural difference between controls and EM individuals by PCoA plotting based on Bray–Curtis dissimilarity data sets at the species level. **(F, G)** Differences in alpha-diversity between the control clusters from EM. **(H)** Peritoneal fluid microbial community structural difference between controls and EM individuals by principal component analysis plotting based on taxonomic data sets at the species level. **(I, J)** Differences in alpha-diversity between the control clusters from EM in PF samples. **(K)** Seven taxa were significantly different between groups. The p value denotes two-tailed Student’s *t*-test. N.S., not significant, *p < 0.05, **p < 0.01.

We applied PCoA to the Bray–Curtis distances of 16S rRNA gene profiles generated from fecal samples collected from the control group and EM group (**Figure 3A**). Differences in microbiome composition between two groups can be observed in the PCoA1 axis (LSD; **Figure 3A**). Compared with the FN group, microbiomes of FE had significantly reduced Shannon and Simpson index-estimated microbial richness (Mann–Whitney U test, p = 0.006 and 0.013, respectively; **Figures 3B, C**). Overall beta-diversity (PCoA) and Shannon (FN: 2.66 ± 0.21, FE: 2.38 ± 0.35) and Simpson (FN 0.88 ± 0.03, FE 0.83 ± 0.07) indices were significantly different between women with or without EM, indicating that a plethora of bacterial taxa contributed to the difference between groups. To probe into the taxonomic composition of the FN and FE samples, top 10 most abundant taxa were widely different among individuals as shown in the bar plot (**Supplemental Figure 1A**). Notably, the most prevalent taxon in stool samples was assigned as *Clostridiales Ruminococcaceae* (average relative abundance: FN 0.21 ± 0.09, FE 0.17 ± 0.11; expressed as mean ± SD). Moreover, *Bacteroidaceae Bacteroides*, *Lachnospiraceae Blautia*, *Clostridiales Lachnospiraceae*, *Faecalibacterium prausnitzii*, and *Bifidobacteriaceae Bifidobacterium* were also among the top 10 most abundant taxa. Ten taxa including *Clostridia Clostridiales*, *Lachnospiraceae Ruminococcus*, *Clostridiales Lachnospiraceae*, and *Ruminococcaceae Ruminococcus* were significantly depleted, while two taxa including *Eggerthella lenta* and *Eubacterium dolichum* were significantly enriched in FE compared with those in the FN (Mann–Whitney U test, all p < 0.05, **Figure 3D**).

### Composition of the Fecal Microbiota Differs Between Early and Advanced Stage EM Patients

We further analyzed the taxonomic composition of the gut microbiome in EM at the early (stage I or II) and advanced stages (stage III or IV). Within the EM group, five patients were diagnosed as early stage and the remaining 16 patients were at the advanced stage (**Supplemental Figure 2A**). Despite that PCoA analysis based on Bray–Curtis distances showed no significant difference at the species, genus, and family levels, we observed a significant difference on axis 3 at the order level (LSD; **Supplemental Figure 2B**).

### Microbiota Composition of the Cervical Mucus Appeared to Be Similar Between Individuals

The microbiota of cervical mucus (CV) was shown to be similar between the control group and EM patients based on beta diversity and alpha diversity (Simpson and Shannon indices) (**Figures 3E–G**). Simpson (CVN 0.44 ± 0.29, CVE 0.39 ± 0.27) and Shannon (CVN 1.13 ± 0.92, CVE 0.86 ± 0.58) indices mildly differ between groups and showed low alpha diversity, indicating that bacterial richness in the CV was low. Taken together, we found that the cervix harbors a low-diversity and low-biomass microbiome, which is in concordance with the standard consensus that cervical mucus is an impermeable elastic barrier against bacteria (McGuckin et al., 2011). The taxonomic profiles of the CVN and CVE samples were mildly different, and the most abundant taxon was assigned as *Lactobacillaceae Lactobacillus* (CVN 0.34 ± 0.38, CVE 0.37 ± 0.45; **Supplemental Figure 1B**; only the top 10 most abundant taxa were shown). None of the taxa of CVE samples appeared to be significantly depleted or increased compared to that of CVN.

### Microbiota Composition of the Peritoneal Fluid Differs Between Control Group and EM Group

The composition of microbiota could not be entirely separated between PFN and PFE samples according to their corresponding diagnosis as shown in PCA plots based on species relative abundance showing the first and second dimensions (**Figure 3H**). Simpson (PFN 0.84 ± 0.31, PFE 0.85 ± 0.25; **Figure 3I**) and Shannon (PFN 3.78 ± 1.58, PFE 3.71 ± 1.43; **Figure 3J**) indices mildly differ between groups. The taxonomic profiles of the PFN and PFE samples were mildly different, and a handful of individuals harbor a distinct microbial composition (**Supplemental Figure 1C**). However, comparison of the relative abundance of OTUs revealed significant differences between the peritoneal fluid microbiota of EM patients and that of controls. A Student’s *t*-test was performed for each taxon between PFN and PFE. Only those markers with a p value below 0.05 were considered significant and shown in the bar plot. Patients with EM had an increased abundance of taxa classified as *Pseudomonadaceae Pseudomonas*, *Prevotellaceae Prevotella*, and *Xanthomonadaceae Luteimonas*, among others, and *P. Pseudomonas* was previously reported as an EM-related pathogen in the peritoneal cavity (all p < 0.05, **Figure 3K**). On the other hand, certain gram-positive bacteria, including *Actinomycetales Microbacteriaceae*, *Lactobacillus iners*, and *Microbacteriaceae Cryocola*, were significantly depleted in the EM group.

### Crucial Bacteria of Gut, CV, and PF Microbiome Related to EM, Based on Random Forest Feature Selection

**Figure 4:**
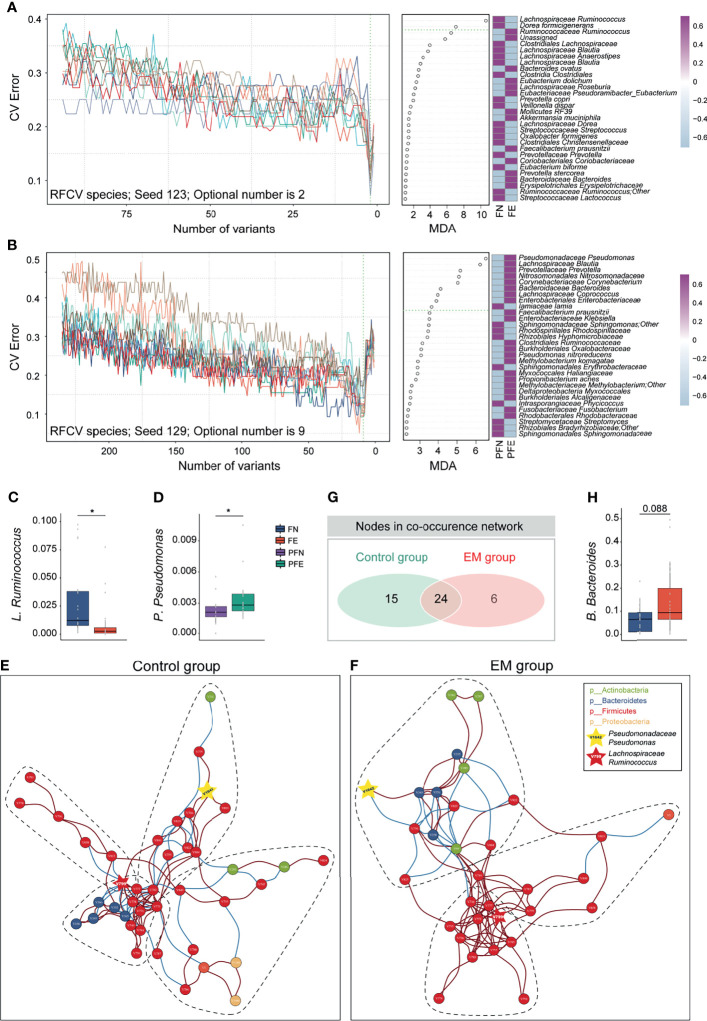
Ecological relationships between microbiota within different habitats. **(A)** Random forest and five-fold cross validation (RFCV) models to predict fecal biomarker in EM. **(B)** RFCV models to predict biomarker in peritoneal cavity. **(C, D)** Box plots illustrated the relative abundance of *Lachnospiraceae Ruminococcus* and *Pseudomonadaceae Pseudomonas* between groups. **(E, F)** The co-occurrence networks depicting commensal correlation of gut bacteria and *P. Pseudomonas* of control and EM, respectively. Nodes are colored by which phylum it belongs to. Based on Spearman correlation coefficients, the edges between each pair of nodes are colored by red (positive correlation, p < 0.05) or blue (negative correlation, p < 0.05). **(G)** Venn diagram shows the number of overlap nodes. **(H)** Box plots illustrated the relative abundance of *Bacteroidaceae Bacteroides* between groups; box boundaries show quartile. The p value denotes Mann–Whitney U test. *p < 0.05.

Knowing that much difference exists in the gut microbiota and peritoneal microenvironment, we presume that microbial markers of EM may exist. We then sought to identify diagnostic biomarkers for EM based on microbiota profiles of paired samples from the gut, cervix, and peritoneal fluid from our subjects. Ten trials of fivefold cross-validation of the random forest classifier models between 20 control group subjects and 21 EM patients were constructed respectively to identify potential bacteria of different body sites (**Figures 4A, B** and **Supplemental Figures 3**–**5**). Within every cross-validation in each trial, 80% of the data were randomly selected as training data and the remaining 20% as the test set to evaluate the performance. The process of random splitting was repeated 500 times, and *Lachnospiraceae Ruminococcus* (FN 0.03 ± 0.03, FE 0.01 ± 0.02; **Figure 4C**) was constantly being defined as predictive in the gut microbiome, while *Pseudomonadaceae Pseudomonas* (PFN 0.0021 ± 0.0013, PFE 0.0034 ± 0.0020; **Figure 4D**) was selected as a key biomarker in peritoneal cavity.

### Network Analysis Shows Co-Abundance of Gut Pathogens and Microbial Marker in Peritoneal Cavity

To gain a deeper insight into the correlative bacterial populations between bacterial taxa in the gut and microbial marker from peritoneal cavity, co-occurrence and anti-occurrence network analyses using a robust greedy algorithm for similarity or dissimilarity were employed. The bacterial correlations among the gut microbiota and *Pseudomonadaceae Pseudomonas* with and without EM were apparently dissimilar (**Figures 4E, F**). In the control group, four subcommunities with 39 species (nodes), 66 positive connections, and 36 negative connections (edges) were retained under a 0.3 correlation cutoff (Spearman’s correlation coefficient > 0.3 or < -0.3, q < 0.05, **Figure 4E**). Under the same condition, three subcohorts with 30 species (nodes), 78 positive connections, and 18 negative connections (edges) can be seen in the co-occurrence networks of the EM group (**Figure 4F**). Of note, *Lachnospiraceae Ruminococcus* (V799) and *Pseudomonadaceae Pseudomonas* (V1642) can be found in both networks. To further elucidate the difference between microbial networks under different EM statuses, we contrasted the number of nodes and edges in the EM group with that of the control group. Despite having a few exclusive nodes respectively, 24 nodes were overlapped (**Figure 4G**). In the co-occurrence network of the EM group, *Clostridiales Clostridiaceae* (V750) was negatively correlated with *Pseudomonadaceae Pseudomonas* (V1642) and concordantly had a significantly lower microbial abundance compared to the control group (Mann–Whitney U test, p < 0.05; **Figures 3D, K**). Nevertheless, *Bacteroidaceae Bacteroides* (V349) positively correlated with *Pseudomonadaceae Pseudomonas* (V1642) and exhibited a higher relative abundance compared to that of the control group (Mann–Whitney U test, p = 0.088; **Figures 4F, H**).

### Potential for Distinguishing EM Patients From Control Group Based on Microbial Markers

**Figure 5:**
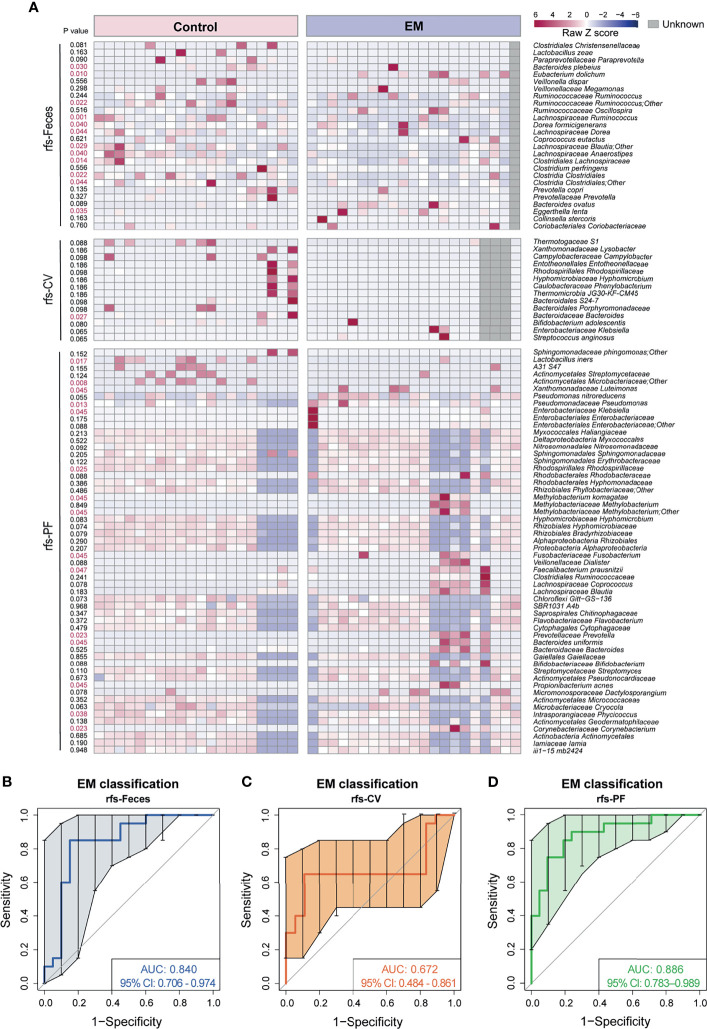
Novel models trained on random forest selected taxa to predict EM. **(A)** Heatmap of random forest selected species union. **(B–D)** Curves of receiver operating characteristics for classification of EM based on random forest selected fecal taxa, cervical mucus taxa, and peritoneal fluid taxa shown in the heatmap above.

Considering that significantly different microbial compositions and relative abundances for a number of species were identified in EM subjects versus control group individuals, we sought to construct a robust classifier for distinguishing EM patients from the control group with the union of optimal microbial markers (rfs-Feces, rfs-CV, rfs-PF) selected from the 10 trials of random forest and five-fold cross-validation of different body sites (**Figure 5A** and **Supplemental Figures 3**–**5**). The random forest (RF) model based on the selected 26 species from microbiota of feces was trained to classify EM, with an area under the curve (AUC) of 0.840 (95% CI: 0.706-0.974; **Figure 5B**). The RF models based on the selective taxa of CV and PF were also trained individually, resulting in AUC of 0.672 (95% CI: 0.484-0.861; **Figure 5C**) and 0.886 (95% CI: 0.783-0.989; **Figure 5D**). The RF models based on microbial markers of feces and PF performed significantly better than that of CV, suggesting that the model trained on the CV species was not able to differentiate EM.

As a complementary approach for identifying EM biomarkers, an indicator value was also calculated to assess the microbiota at the species level. The indicative species from different body sites were shown in a heatmap with p value on the left side (**Supplemental Figure 6A**). However, RF models trained on the indicative species did not perform well in discriminating EM, with an AUC of 0.738 (IndVs-Feces, 95% CI: 0.570–0.905; **Supplemental Figure 6B**), 0.625 (IndVs-CV, 95% CI: 0.528–0.722; **Supplemental Figure 6C**), and 0.782 (IndVs-PF, 95% CI: 0.631–0.933; **Supplemental Figure 6D**), respectively. Moreover, Venn diagrams displaying the commonly used taxa in EM classifier models between groups showed that nine taxa were shared between rfs-Feces and IndVs-Feces (**Supplemental Figure 6E**) and rfs-CV contained the only taxa in IndVs-CV (**Supplemental Figure 6F**), while six taxa were shared between rfs-PF and IndVs-PF (**Supplemental Figure 6G**). Notably, random forest-selected taxa included most of the indicator species respectively, again validating the robustness of this novel methodology in massive feature selection.

## Discussion

By profiling the peritoneal fluid microbiomes of women with endometriosis at the reproductive age, the current study provides a new clue that microbes in the peritoneal cavity may contribute to establish a pathogenic microenvironment. It should be noted that the retrograde menstruation theory raised by Sampson in 1927 was broadly accepted (Sampson, 1927). To date, 80% women at the reproductive age experience retrograde menstruation, but the prevalence of endometriosis is around 10% (Hickey et al., 2014). It gave us a hint that there may be unknown factors present in the peritoneal cavity that could possibly affect the local environment. Previous studies also demonstrated that the microenvironment of the peritoneal cavity in endometriosis appears to be pro-inflammatory (Osteen et al., 2005) and the peritoneal cavity was proven to be not sterile (Chen et al., 2017). However, investigations into microbes of the peritoneal cavity had few progresses for the difficulty of obtaining peritoneal fluid specimen. Collecting peritoneal fluid from healthy control would be considered highly inappropriate and unapproachable. Most importantly, women with endometriosis may have no symptom, and empirical diagnosis cannot rule out endometriosis. Instead, recruiting women with moderate gynecological diseases, with a root cause not involving peritoneal microenvironment, as control subjects, had allowed the investigation of the difference of the peritoneal microenvironment between EM patients and non-endometriosis subjects. On the other hand, symptoms of endometriosis often onset at the early age followed with a 4–11-year delayed diagnosis was constantly reported worldwide (Nnoaham et al., 2011; Hudelist et al., 2012; Agarwal et al., 2019). Performance of auxiliary diagnosis methods, including but not limited to transvaginal ultrasound (TVS), transabdominal ultrasound (TUS), and magnetic resonance imaging (MRI), varies between studies depending on multiple factors, such as radiologists’ experience and the fundamental structures of different facilities (Guerriero et al., 2013; Wildenberg et al., 2016; Guerriero et al., 2018). A solid diagnosis of endometriosis requires surgical visualization with histological verification (Rösner and Böger, 1979); however, it is unrealistic for all women with suspected endometriosis to receive an invasive surgery while the recurrence rate is high and the surgery could possibly damage the reproductive system (Ahn et al., 2017). In this regard, searching for a valid but non-invasive biomarker is an urgent need and of great benefit to women who do not wish to undergo a surgical operation. Studies more recently suggested that endometriosis may originate with immunology perturbations (Symons et al., 2018), which is in association with microbiome (Opdenakker et al., 2016). Microbiota, proved to play multiple critical roles within a host (Fan et al., 2021), is also implicated to be correlated with endometriosis (Yuan et al., 2018; Ata et al., 2019; Chadchan et al., 2019; Ni et al., 2020). Accordingly, establishing the relationship between the microbiota of different body sites for women with or without endometriosis and interrelationship of microbiota with different endometriosis stages may give us an extra knowledge on the susceptibility and aggravation of endometriosis. Based on the next-generation sequencing (NGS) technique and/or bacterial cultivation, investigators suggested that peritoneal cavity and female genital tract (FGT) harbor unique microbial communities (Chen et al., 2017; Akiyama et al., 2019; Ata et al., 2019). Moreover, microbial colonization of the peritoneal cavity could originate from the gastrointestinal tract or the lower female genital tract *via* bloodstream or ascension (Rescigno et al., 2001; Baker et al., 2018). Herein, using matched sampling, we collected feces, cervical mucus, and peritoneal fluid from 20 non-endometriosis and 21 endometriosis individuals aiming to reveal the correlation between endometriosis and microbiota from different body sites. Primarily, we demonstrated systemic analysis and provided clues in accessing the association between microbiota and endometriosis across three body sites. In addition, we have elucidated the aberrant taxonomic composition in feces and peritoneal fluid of endometriosis versus that of the controls, but conversely, cervical microbiota differs slightly among individuals. Most importantly, we have identified two microbial biomarkers that mightily contributed in differentiating endometriosis and complex co-occurrence network elucidating potential coexistence patterns of microbial communities. Eventually, robust endometriosis classifiers were constructed based on endometriosis-associated bacteria of feces and peritoneal fluid, with AUC = 0.840 and AUC = 0.886, respectively. Moreover, the results suggest that gut microbiota performs better than cervical microbiota in early diagnosis of endometriosis. The idea of electing endometriosis-relative taxa for endometriosis classifier establishment may allow us to diminish the confounding factors and precisely identify endometriosis patients by evaluating personal gut microbiota.

Decades of research into the etiology and pathogenesis of endometriosis have partially elucidated the underlying mechanisms involved in disease occurrence and progression, whereas endometriosis-related risk factors are still under exploration. Although ectopic endometrium tissue is mainly located in the peritoneal cavity, the microbiome from the gut and cervix might still have a role in modulating endometriosis (Ata et al., 2019; Perrotta et al., 2020); however, there is no study ever investigating the “cross talk” between the microbiome from the gut, cervix, and peritoneal fluid. Thus, by comparing α- and β-diversity as well as microbial profiling between microbiomes derived from different body sites, we identified a distinct inter-habitat microbial composition and potential correlation between different body sites. Species of the *Enterobacteriaceae Klebsiella*, especially *K. pneumoniae*, were reported to be opportunistic pathogens through producing extended-spectrum beta-lactamase and have the potential to cause spontaneous bacterial peritonitis (Jae et al., 2014). Another gram-negative bacterium of the family of *Enterobacteriaceae*, *Salmonella*, was able to reach the spleen after oral administration, suggesting the existence of penetration of the gut mucosa by pathogens (Rescigno et al., 2001). Interestingly, in the current study, *Enterobacteriaceae Klebsiella* was enriched in the cervix of endometriosis compared to the control group, indicating that *E. Klebsiella* may migrate to the peritoneal cavity and modulate the microenvironment by causing inflammation. On the other hand, *Prevotella* spp. are common oral, vaginal, and gut commensals, reported to be a potential mediator in modulating the body mass index and hormone biosynthesis. *Bacteroidales S24-7* (recently classified as *Muribaculaceae*) were identified as potential mucus degraders in several previous studies (Berry et al., 2015; Lagkouvardos et al., 2019; Lee et al., 2019), therefore impeding pathogens that utilize mucus-derived sugars as crucial nutrients (Pereira et al., 2020). In this study, we observed pronounced abundance of *Bacteroidales S24-7* in peritoneal fluid of the control group. Likewise, *Enterobacteriaceae Klebsiella*, *Prevotellaceae Prevotella*, and *Bacteroidales S24-7* together with the remaining 16 microbial taxa shared by feces, cervical mucus, and peritoneal fluid may also have a role in modulating the microenvironment of the peritoneal cavity *via* metabolic function and/or penetration depending on the milieu. Thus, the current study provides a clue for future investigation into the association between the key commensal microbes at different body sites in the progression of endometriosis.

Up to now, little attention has been paid to key microorganisms and their function related to endometriosis. Specific negative associations with metabolic health problems, e.g., cardiovascular or inflammatory diseases, were detected for several genera, including *Roseburia* (*Clostridiales* order, *Clostridia* class), a genus of butyrate-producing colonic bacteria (Louis and Flint, 2009; Pichler et al., 2020). Reduction of butyrate eventually results in a disturbed integrity of epithelial cells and consequently in an increased colonic permeability (Kinoshita et al., 2002). In this study, the pattern of gut microbiota has been comprehensively elucidated and *Clostridiales Clostridia*, *Clostridiales Lachnospiraceae*, and *Lachnospiraceae Ruminococcus* were significantly reduced in FE compared with FN. This indicates that certain alterations of microbiome and metabolites in the gut may be involved in the regulation of physiological function. Unfortunately, no studies have demonstrated the interaction between these microorganisms and endometriosis, which are worth more focus and further exploration. Moreover, several of the taxa identified to be distinct between groups in this study are known to exert negative effects in intestinal homeostasis. Patients with Crohn’s disease are reported to harvest a decreased abundance of several genera, including *Ruminococcus* and *Dorea*, within the families of *Ruminococcaceae* and *Lachnospiraceae* (Gevers et al., 2014). Interestingly, in this study, *Ruminococcaceae Ruminococcus* and *Lachnospiraceae Dorea* were remarkably reduced in FE versus FN. Others, such as *Eggerthella lenta*, were significantly increased in FE, while being reported to be potentially harmful (Fattorusso et al., 2019), reminding us that the loss of protective microbes could be the triggering factor in exacerbating the proliferation of certain potential pathogens (Looft and Allen, 2012), inducing systemic inflammation. In conclusion, feces of endometriosis individuals were aberrant from that of the control subjects and beta diversity analysis has pinpointed endometriosis status as a potent factor in distinguishing FE from FN. Although the ectopic endometrium is mostly located in the peritoneal cavity, the microbial difference of peritoneal fluid between groups is not as prevalent as that of feces in our study. Endometriosis is characterized to be an inflammatory-like disease, and pro-inflammatory cytokines were enriched in PF of women with endometriosis (Dziunycz et al., 2009). Beta diversity analysis implies that PFE is significantly different from PFN, but the EM group did not separate from the control group completely. One possible explanation could be that peritoneum mesothelial cells continuously produce peritoneal fluid, followed by reabsorption through the large surface area of the peritoneum and removal of pathogens and cells ascending from the female genital tract (Blackburn and Stanton, 2014; Capobianco et al., 2017), resulting in a rather stable microenvironment. In the endometriosis group, we observed increased abundance of Gram-negative bacteria, including *Pseudomonadaceae Pseudomonas* and *Prevotellaceae Prevotella*, with the potential of releasing lipopolysaccharide (LPS) (Raia et al., 2005), which can activate macrophages and further induce secretion of a variety of local products, enhancing the proliferation of endometriotic stromal cells (Sakamoto et al., 2003).

Knowing that much difference exists in the gut microbiota and peritoneal microenvironment, we presume there might be microbial markers of endometriosis. Animal studies have elucidated that the gut microbiota might have a role in promoting endometriosis disease progression, and communication between the host and gut microbiome might be bidirectional (Yuan et al., 2018; Chadchan et al., 2019; Ni et al., 2020). In numerous human studies, noninvasive peripheral markers have been explored in blood, tissue, feces, cervical mucus, peritoneal fluid, and urine from endometriosis patients compared with non-endometriosis subjects: imaging, proinflammatory cytokines, miRNAs and metagenome, etc. (Upson et al., 2014; Wessels et al., 2015; Nematian et al., 2018). However, these research results are inconsistent possibly due to differences in study design, biologic sample collection, storage, data analysis workflow, and defecation time. Owing to ethical constraints, we adopted the least interferential protocol in collecting peritoneal fluid, which is internally consistent with Chen et al. (Chen et al., 2017). Moreover, all fecal samples were collected 1 to 3 days before the laparoscopy procedure in order to eliminate the variation which could be possibly caused by invasive surgery. Notably, we highlighted the unique bacterial differences with biomarker potential that are found in the feces and peritoneal fluid. The reduced level of *Lachnospiraceae Ruminococcus* in the gut and increased abundance of *Pseudomonadaceae Pseudomonas* in peritoneal cavity are closely associated with endometriosis, providing a hint on future diagnosis and therapy. Interestingly, *Ruminococcus faecis* was reported to be positively correlated with SCFA production, especially the biosynthesis of butyrate (Jin et al., 2019), believed to counteract gastrointestinal cancer and inflammation. In addition, formation of propionate, a type of SCFAs with potential health-promoting effects, was accomplished by *Ruminococcus obeum* (in the family of *Lachnospiraceae*) in the human large intestine (Jin et al., 2019). Taking into account the aforementioned specificities of SCFAs, we hypothesize that the reduction of *L. Ruminococcus* may result in decreased concentration of protective metabolites in the human intestine, leading to the development of diseases. On the other hand, arylsulfatase from *Pseudomonas aeruginosa* was able to enhance the steroid sulfatase activity (Uduwela et al., 2018), which is capable of catalyzing sulfated steroid precursors to the free steroid. Enhanced steroid sulfatase activity included catalyzing estrone sulfate to estrone, a weak estrogen. In previous results, prolonged exposure to estrogen was reported to be an environmental toxin correlated with the occurrence of endometriosis (Huhtinen et al., 2012). In summary, enriched *P. Pseudomonas* in the peritoneal cavity of endometriosis patients may be a result in the increased level of estrogen in the microenvironment, thereby increasing the estradiol concentration locally and thus exacerbating the proliferation of the ectopic endometrium (Huhtinen et al., 2012).

Despite the emerging evidence for a correlation of *P. Pseudomonas* and endometriosis (Akiyama et al., 2019; Hernandes et al., 2020), we cannot ignore the importance of bacterial migration and microbial communities themselves as a disease promoter. To peek into the relationship across gut flora and the potential pathogen, *P. Pseudomonas*, co-occurrence networks were built, in which both *L. Ruminococcus* and *P. Pseudomonas* were shown to be within certain communities. The commensal community, instead of an independent taxon, is also an intriguing possibility in disease occurrence. This observation emphasizes the need to unite opportunistic commensals in future research. The knowledge of the community composition of microbiome may precipitate strategies in mitigating the debilitating symptom of endometriosis and lead to the development of probiotics which has the potential to be an additional treatment for endometriosis patients.

Attempting to construct novel endometriosis classifiers, a hypothesis-free machine-learning strategy was adopted based on the taxa selected in 10 trials of RFCV from three body sites respectively, from which we concluded that the endometriosis classifier based on fecal or peritoneal fluid microbiota performs much better and steadily compared with that of cervical mucus. Interestingly, a similar trend of accuracy was shown in models built on the indicator species, with a mass overlap with random forest-selected taxa, indicating that microbiota in feces and peritoneal fluid is of greater importance in predicting endometriosis. Moreover, either obtaining cervical mucus or peritoneal fluid is not friendly to traditional concept and would acquire the guidance of professional physicians or surgeons. This suggests that fecal microbiota is of great importance in the classification of endometriosis compared to that of cervical mucus and peritoneal fluid, laying the basis for the usage of the endometriosis classifier model based on RFCV-selected taxa in subsequent studies. However, a limitation for the accuracy of models may be the handful of population included in the study; thus, validation in larger cohorts is of great necessity and urgent need. In previous studies, microbiota of cervical mucus was reported to have the potential in identifying endometrial cancer, endometritis, or infertility due to endometriosis (Cicinelli et al., 2008; Walther-António et al., 2016; Chen et al., 2017). Considering that the endometriosis classifier model based on the microbiota of cervical mucus presents low predictive validity in our study, we cannot exclude the possibility that disease cohorts, regional and individual differences in microbiota profiles, and methodological differences in the study design contributed to the taxonomic results.

Taken together, dysbiosis of the gut microbiota and altered microbial composition in the peritoneal cavity may together contribute to the development of endometriosis. The presence of *P. Pseudomonas* may be a potential risk factor in peritoneal cavity, and the depletion of *L. Ruminococcus* in the gut might be a biomarker for endometriosis. Whether endometriosis-related taxa and their intracorrelation or intercorrelation structure are causally linked to different clinical stages of endometriosis remains to be studies, but these findings clearly urge us to consider microbiota alteration in gut flora for novel diagnostic and prognostic research. Most importantly, investigation of the potential effect of *P. Pseudomonas* in the peritoneal microenvironment and elucidation of the underlying mechanism are emerging.

### Limitation of the Study

In the current study, we have provided important insights into the microbial structure across three body sites by obtaining paired samples. Although we have demonstrated that the potential pathogen *P. Pseudomonas* may contribute to the pathogenesis of endometriosis in peritoneal cavity, the control group we recruited in this article was diagnosed with benign gynecology disease, but the root cause does not include peritoneal microenvironment. Subsequent work that utilizes the microbial culture technique may specify the specific strain of potential pathogen *P. Pseudomonas* and elucidate the pathogenesis mechanism with *in vivo* and *in vitro* experiment.

## Materials and Methods

### Participant Enrollment

From June 2019 to October 2019, 1,200 women were admitted to Zhujiang Hospital of Southern Medical University in Guangzhou, China, due to gynecological diseases. At least two gynecologists independently screened and assessed the condition of the participants strictly according to the inclusion and exclusion criteria. Finally, our study included 41 patients (median age 37 years; range 18–52 years) after two rounds of screening. Prior to the experiment, all participants have been informed of the risks and benefits of the experiment and provided written informed consent, and we have obtained their approval. All study-related protocols were approved by the Southern Medical University Clinical Research Ethics Committee and registered at clinicalTrials.gov (NCT05086484).

### Sample Collection

Cervical mucus and stool were collected from 21 women with EM and 20 control group participants 1–3 days before undergoing laparoscopy. Participants were given a fecal sample collection kit and were instructed how to use it and to return the sample to the gynecologists right after the defecation. Cervical mucus was drawn from the cervical canal with sterile swabs in the process of a gynecological examination (without any prior disturbance; carefully avoid contamination from vaginal). Peritoneal fluid was obtained from participants undergoing a diagnostic laparoscopy. All specimens were rapidly processed upon receipt in a laboratory and transferred to -80°C to be stored until RNA extraction.

### 16S rRNA Gene Sequencing

The ZR Fecal RNA Kit (Zymo Research, United States) was used for all samples’ total RNA extraction according to the manufacturer’s specifications. The V4 regions of the 16S rRNA gene were amplified and sequenced on the Ion Torrent S5 platform. The primers used in sequencing were 341F 5′-CCTAYGGGRBGCASCAG and 806R5′-GGACTACNNGGGTATCTAAT. All paired-end files are generated from the Ion Torrent S5 platforms, following merging, removal of barcodes and primers, filter by VSEARCH, and quality control using the QIIME 1.91 workflow (Caporaso et al., 2010; Quince et al., 2016). A total of 122 samples were subscribed for 16s rRNA gene sequencing and obtaining an average of 65,105 reads (min: 30,081, max: 76,005, median: 67,606) per sample. With a 97% similarity threshold, merged clean amplicons were clustered into operational taxonomic units (OTUs), following the “Open-Reference” clustering approach. The RDP classifier against the Greengenes ver. 13.5 database were utilized for taxonomy profiling (McDonald et al., 2012). For all taxonomic data submitted in the subsequent analysis, low abundant taxa (mean relative abundance < 0.1%; prevalence < 10% within each group) were recognized as interfering elements and decontaminated.

### Bioinformatics Analysis

Shannon and Simpson’s indices and the number of taxa remained after decontamination was used to assess the alpha-diversity of the communities. Beta diversity compares feature dissimilarity between each pair of samples by computing Bray–Curtis and Binary–Jaccard. Prior to statistical analysis, a Shapiro–Wilk test was conducted to verify the normality of measurements. Unpaired Student’s *t*-test or Mann–Whitney U test was used for quantitative variables depending on the distribution of the variables. Non-parametric comparisons among subjects between all groups were assessed using the Kruskal–Wallis test for mean values. Significant differences are indicated as *p < 0.05, **p < 0.01, and ***p < 0.005, and N.S. stands for not significant. Principal coordinate analysis (PCoA) was performed with “ggplot2” and “vegan” of R. With the “randomForest” R package, five-fold cross-validation was performed upon a random forest model, constantly setting ntree = 1,000 to estimate the importance score of each taxon. Eventually, 6 million decision trees were generated at the species level with the “randomForest” package of R to identify the significantly differentiating OTUs separating the healthy controls from EM samples. The heatmaps were constructed using the “pheatmap” package, and the area under the receiver operating characteristic curve (AUC) was calculated in logit models with “pROC” of R. Spearman’s correlation coefficient and fast-greedy modularity optimization algorithm assessed bacterial networks at the species level and determined subgroups containing microbial markers in the co-occurrence network based on the “igraph” package.

## Data Availability Statement

The original contributions presented in the study are publicly available in NCBI under accession number PRJNA753920.

## Ethics Statement

The studies involving human participants were reviewed and approved by the Southern Medical University Clinical Research Ethics Committee. The patients/participants provided their written informed consent to participate in this study.

## Author Contributions

LX and YM designed the experiment and acquired grants. LH, BL, ZL, and ZC collected and analyzed the data. LH drafted the manuscript. YW, DP, XF, and HZ collected samples. All authors contributed to the article and approved the submitted version.

## Funding

This work was supported by the ‘GDAS’ Project of Science and Technology Development (Grant Nos. 2021GDASYL-20210102003, 2018GDASCX-0102) to LX, National Natural Science Foundation of China (Grant No. 81701418), the College Students’ Innovative Entrepreneurial Training (Grant No. S202112121053), and the Scientific Research Enlightenment Program of Southern Medical University to YM. This work was also supported by the Guangdong Basic and Applied Basic Research Foundation (Grant No. 2020B1515020046) and by the National Natural Science Foundation of China (Grant Nos. 81900797, 82072436) to LX.

## Conflict of Interest

The authors declare that the research was conducted in the absence of any commercial or financial relationships that could be construed as a potential conflict of interest.

## Publisher’s Note

All claims expressed in this article are solely those of the authors and do not necessarily represent those of their affiliated organizations, or those of the publisher, the editors and the reviewers. Any product that may be evaluated in this article, or claim that may be made by its manufacturer, is not guaranteed or endorsed by the publisher.

## Supplementary Material

Supplementary Figure 1 is related to **Figure 2**. **(A–C)** Cumulative bar charts of the most abundant taxa at phylum level. Samples are ordered according to increasing relative abundances of the most abundant species within each group.

Supplementary Figure 2 is related to **Figure 3**. **(A)** Number of patients with different endometriosis stages, according to revised American Society for Reproductive Medicine (rASRM), were presented in the bar plots. **(B)** PCoA of microbiota from feces in early-stage and late-stage EM patients based on Bray-Curtis dissimilarity at order level. Samples collected from patients of early stage are significantly different from advanced stage samples (axis 3, accounted for 9.65% variance).

Supplementary Figure 3 is related to **Figure 4**. Ten trails of random forest and 5-fold cross validation models to predict fecal biomarker in EM.

Supplementary Figure 4 is related to **Figure 4**. Ten trails of random forest and 5-fold cross validation models to predict cervical mucus biomarker in EM. The optimal model was exhibit in the top, however the optimal 5 taxa elected as microbial marker were similar between groups.

Supplementary Figure 5 is related to **Figure 4**. Random forest and 5-fold cross validation models to predict peritoneal fluid biomarker in EM.

Supplementary Figure 6 is related to **Figure 5**. Models trained on indicator species to predict EM. **(A)** Heatmap of indicator taxa defined by the indicator value. **(B–D)** Curves of receiver operating characteristics for classification of EM based on indicator taxa shown in the heatmap above. **(E–G)** Venn diagrams show the overlap taxa between random forest selected species and indicator taxa of different body sites.
