# Supplementary figures and images for: Gut Microbiota Exceeds Cervical Microbiota for Early Diagnosis of Endometriosis
Source: Front Cell Infect Microbiol. 2021 Dec 7;11:788836. doi: 10.3389/fcimb.2021.788836 (PMC8688745; doi:10.3389/fcimb.2021.788836)

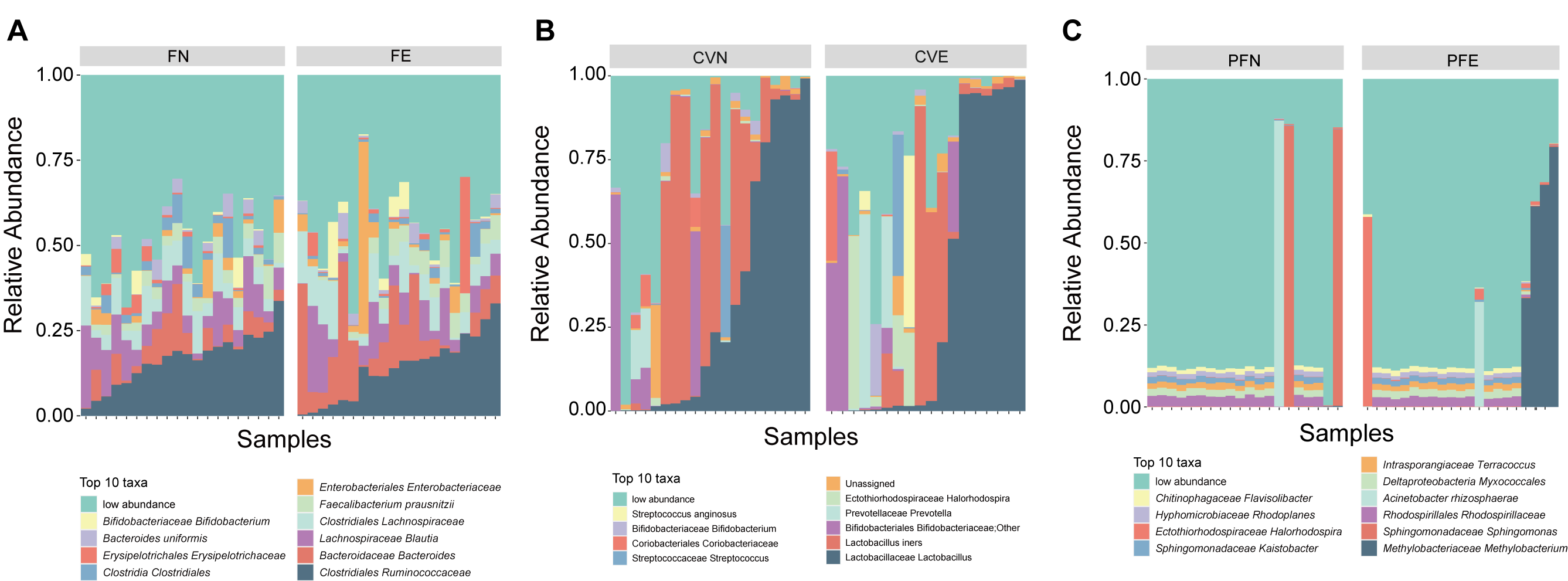

Supplement: Supplementary Figure 1 — is related to Figure 2 . (A–C) Cumulative bar charts of the most abundant taxa at phylum level. Samples are ordered according to increasing relative abundances of the most abundant species within each group. [file Image_1.tif]

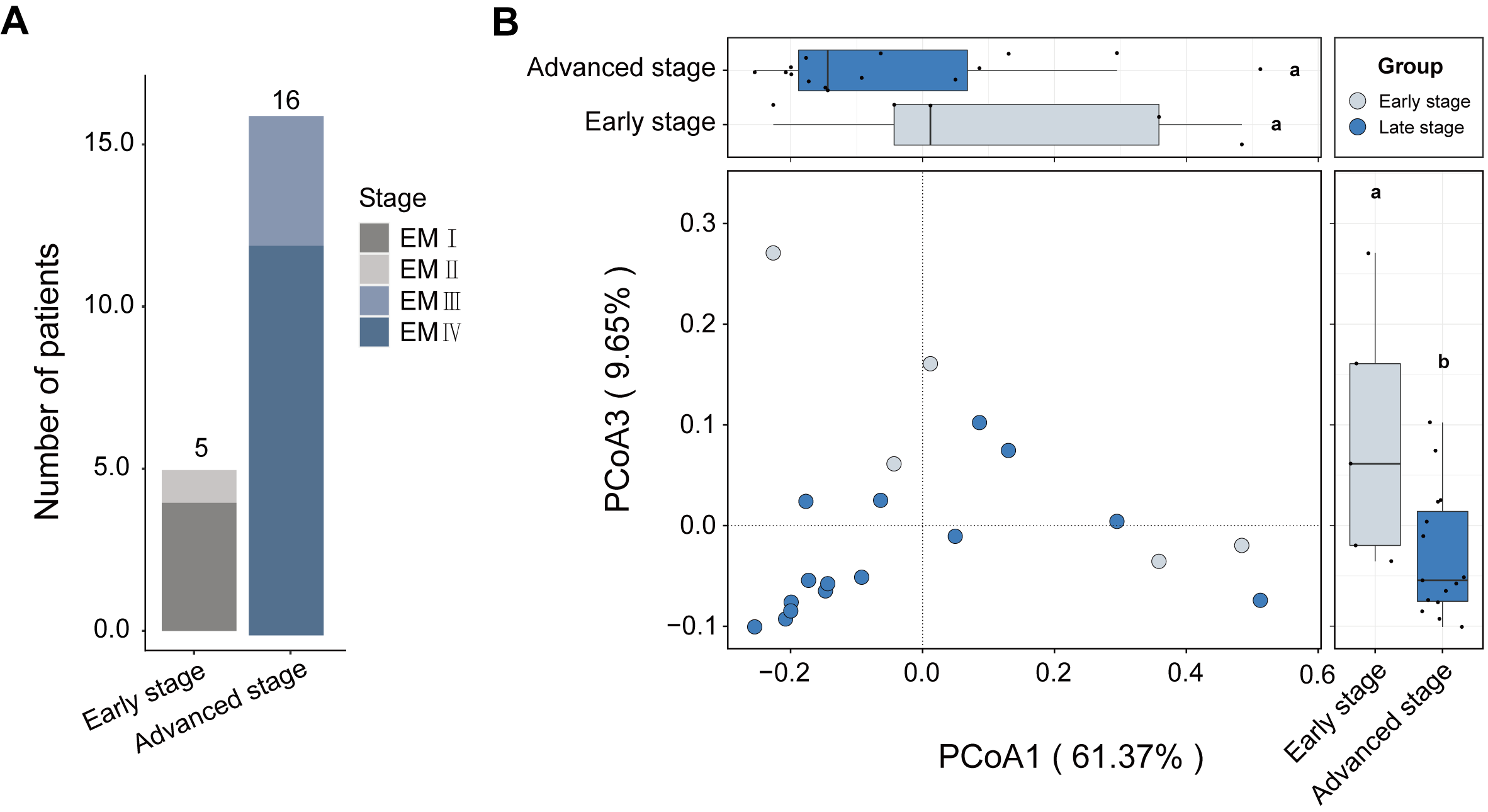

Supplement: Supplementary Figure 2 — is related to Figure 3 . (A) Number of patients with different endometriosis stages, according to revised American Society for Reproductive Medicine (rASRM), were presented in the bar plots. (B) PCoA of microbiota from feces in early-stage and late-stage EM patients based on Bray-Curtis dissimilarity at order level. Samples collected from patients of early stage are significantly different from advanced stage samples (axis 3, accounted for 9.65% variance). [file Image_2.tif]

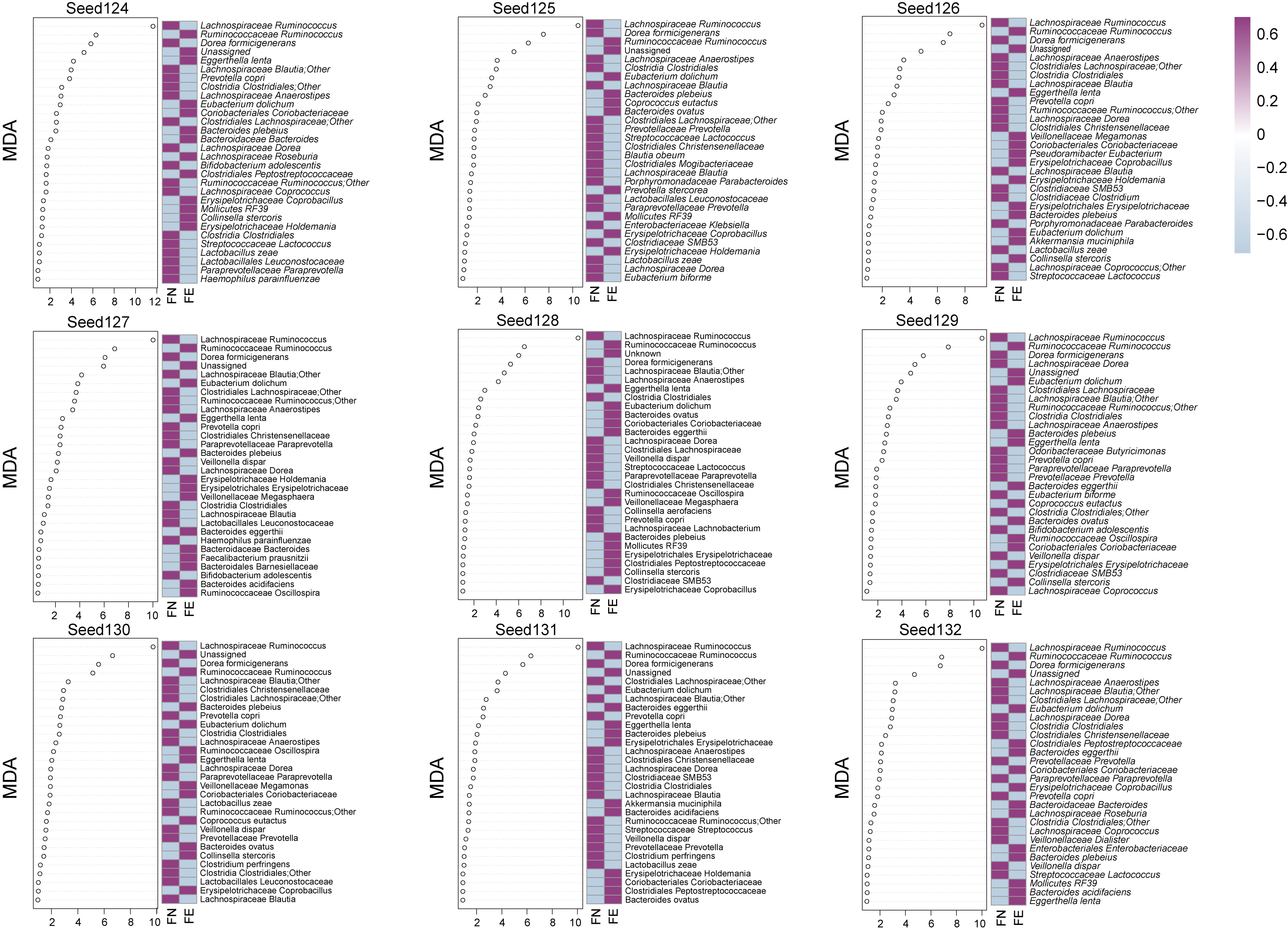

Supplement: Supplementary Figure 3 — is related to Figure 4 . Ten trails of random forest and 5-fold cross validation models to predict fecal biomarker in EM. [file Image_3.tif]

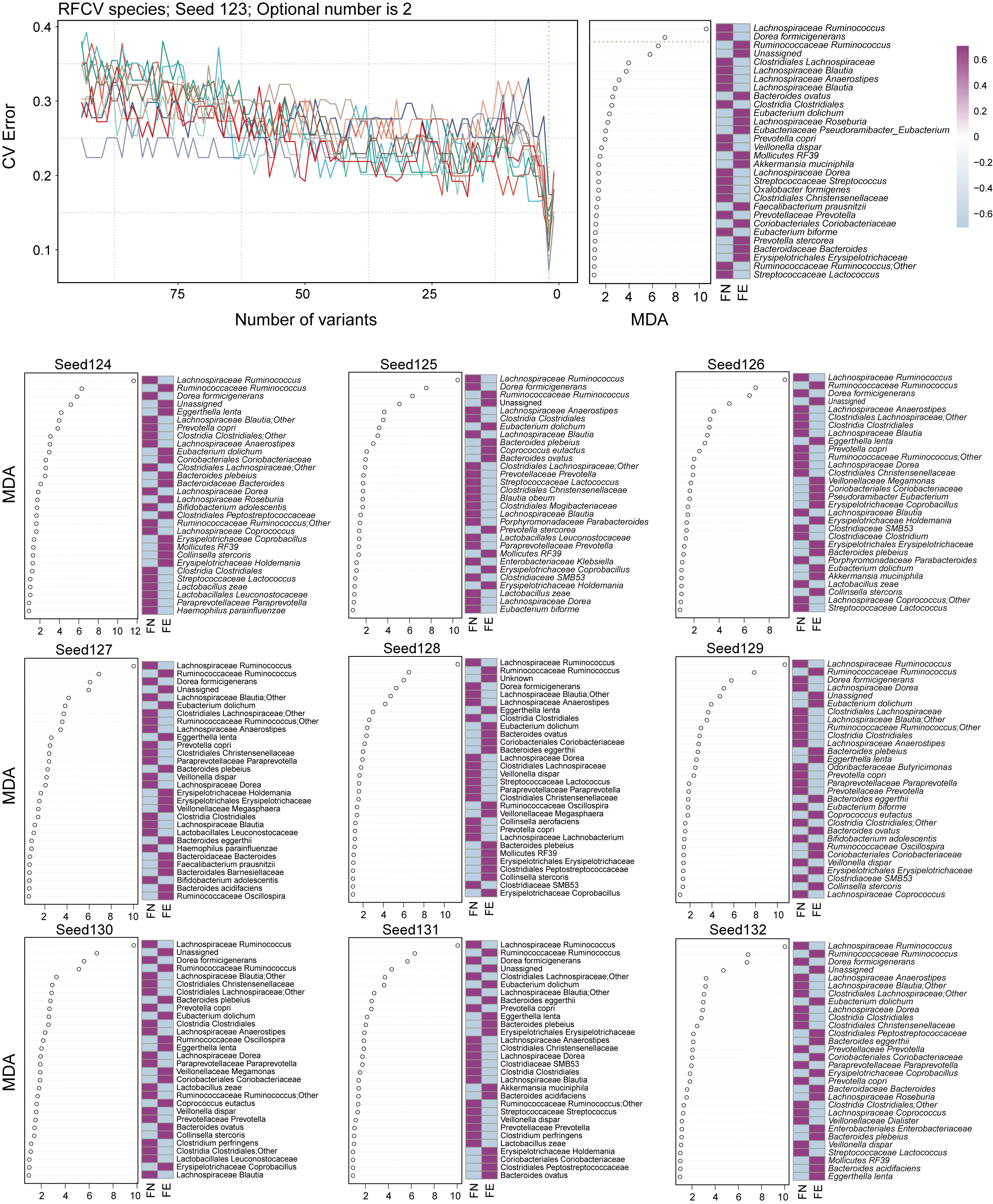

Supplement: Supplementary Figure 4 — is related to Figure 4 . Ten trails of random forest and 5-fold cross validation models to predict cervical mucus biomarker in EM. The optimal model was exhibit in the top, however the optimal 5 taxa elected as microbial marker were similar between groups. [file Image_4.tif]

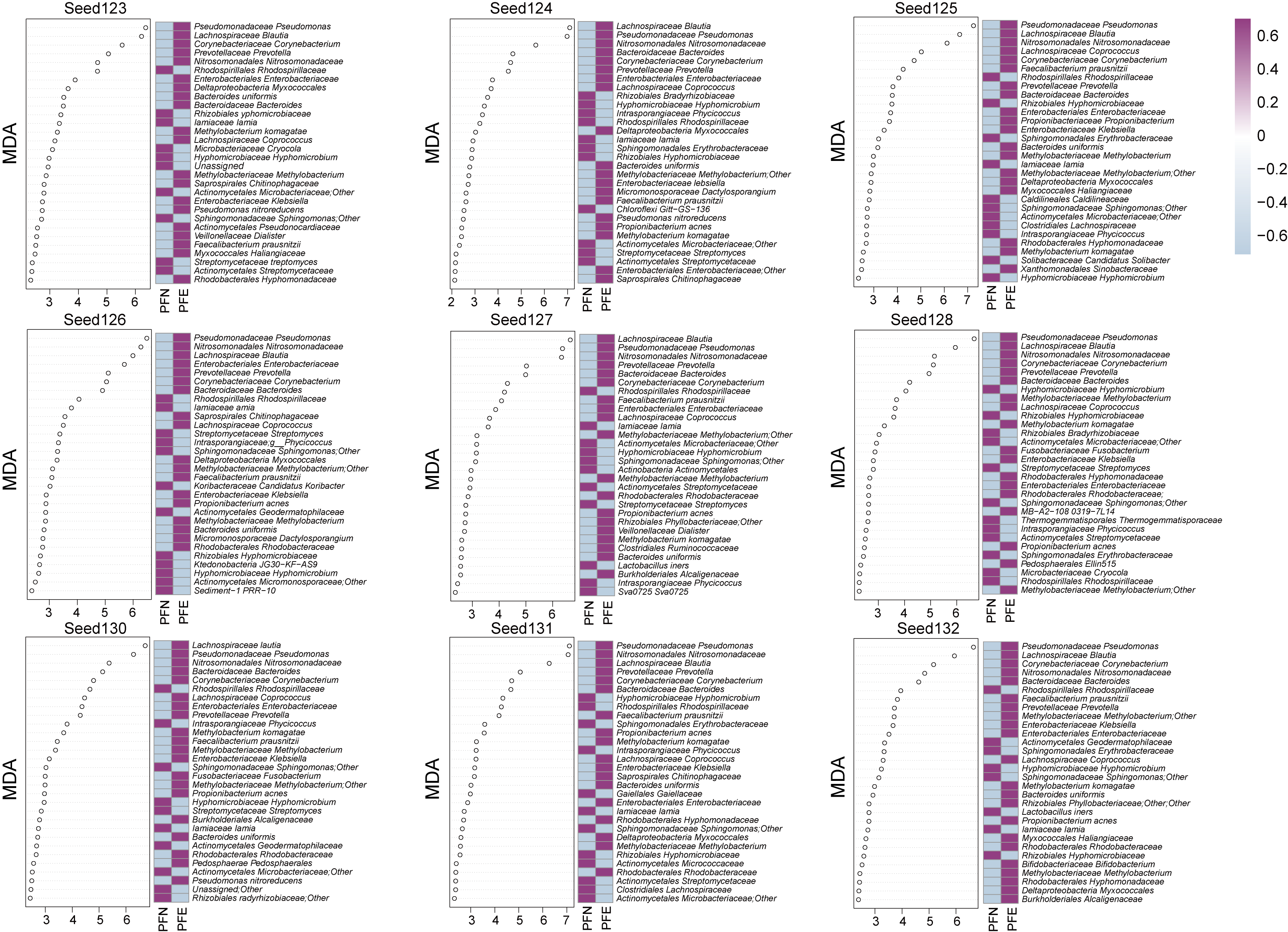

Supplement: Supplementary Figure 5 — is related to Figure 4 . Random forest and 5-fold cross validation models to predict peritoneal fluid biomarker in EM. [file Image_5.tif]

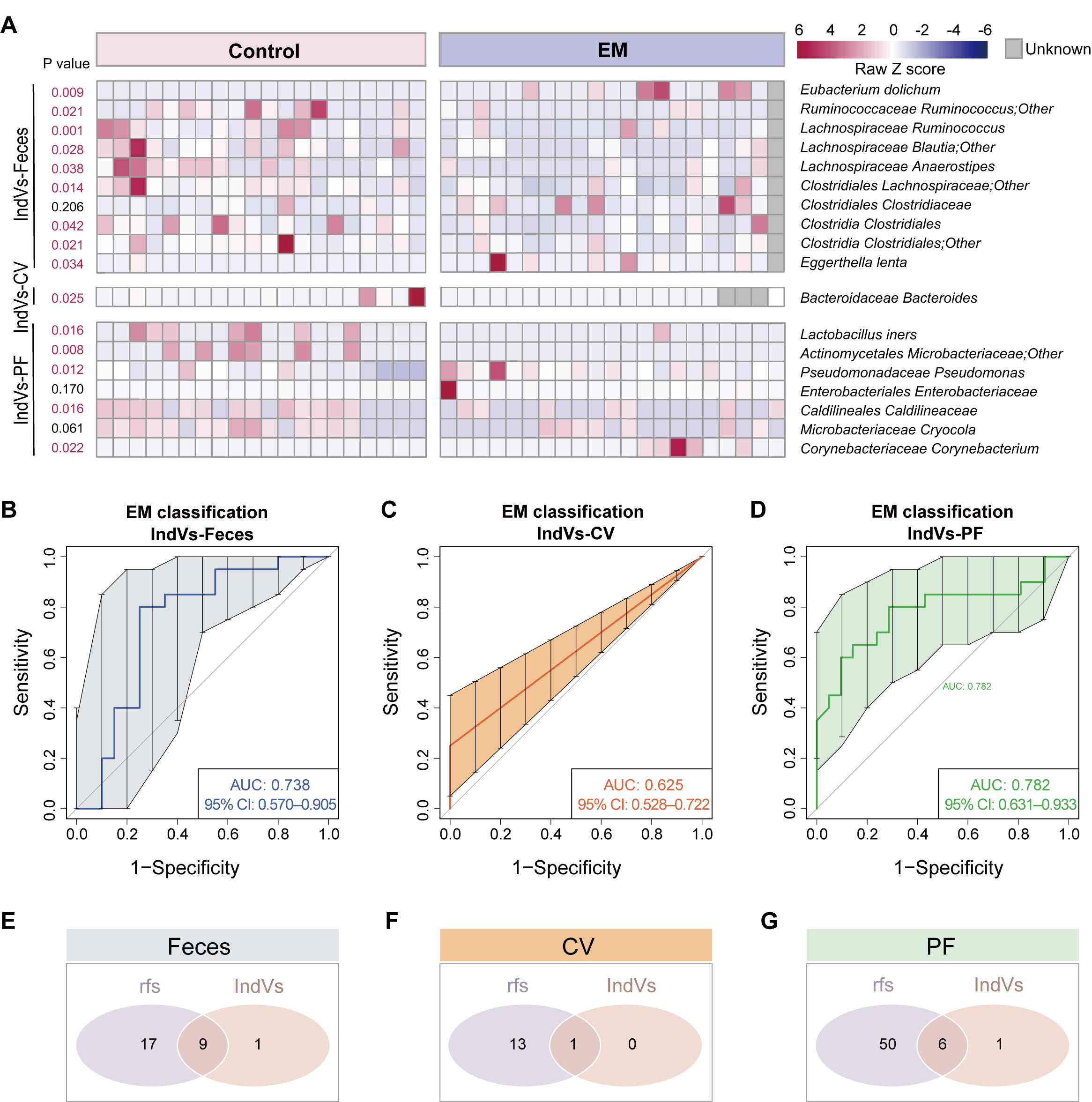

Supplement: Supplementary Figure 6 — is related to Figure 5 . Models trained on indicator species to predict EM. (A) Heatmap of indicator taxa defined by the indicator value. (B–D) Curves of receiver operating characteristics for classification of EM based on indicator taxa shown in the heatmap above. (E–G) Venn diagrams show the overlap taxa between random forest selected species and indicator taxa of different body sites. [file Image_6.tif]
